# Supplementary material for: Galvanic-Replacement-Assisted Synthesis of Nanostructured Silver-Surface for SERS Characterization of Two-Dimensional Polymers
Source: Sensors (Basel). 2024 Jan 12;24(2):474. doi: 10.3390/s24020474 (PMC10819046; doi:10.3390/s24020474)
Supplement: Supplementary file 1 [file sensors-24-00474-s001.zip › sensors-2784921-supplementary.pdf]

Supporting Information for

Galvanic-Replacement-Assisted Synthesis of Nanostructured Silver-Surface for SERS Characterization of Two-Dimensional Polymers

Wenkai Zhao [1,2,†], Runxiang Tan [1,3,†], Yanping Yang [1], Haoyong Yang [1,2], Jianing Wang [1], Xiaodong Yin [1,2], Daheng Wu [1] and Tao Zhang [1,*]

[1] Key Laboratory of Marine Materials and Related Technologies, Zhejiang Key Laboratory of Marine Materials and Protective Technologies, Ningbo Institute of Materials Technology and Engineering, Chinese Academy of Sciences, Ningbo 315201, China; zhaowenkai@nimte.ac.cn (W.Z.); tanrunxiang@nimte.ac.cn (R.T.); yangyanping@nimte.ac.cn (Y.Y.); yanghaoyong@nimte.ac.cn (H.Y.); wangjianing@nimte.ac.cn (J.W.); yinxiaodong@nimte.ac.cn (X.Y.); wudaheng@nimte.ac.cn (D.W.)

[2] University of Chinese Academy of Sciences, Beijing 100049, China

[3] Key Laboratory of Leather Chemistry and Engineering of the Education Ministry, Sichuan University, Chengdu 610065, China

* Correspondence: tzhang@nimte.ac.cn

† These authors contributed equally to this work.

Supporting Figures

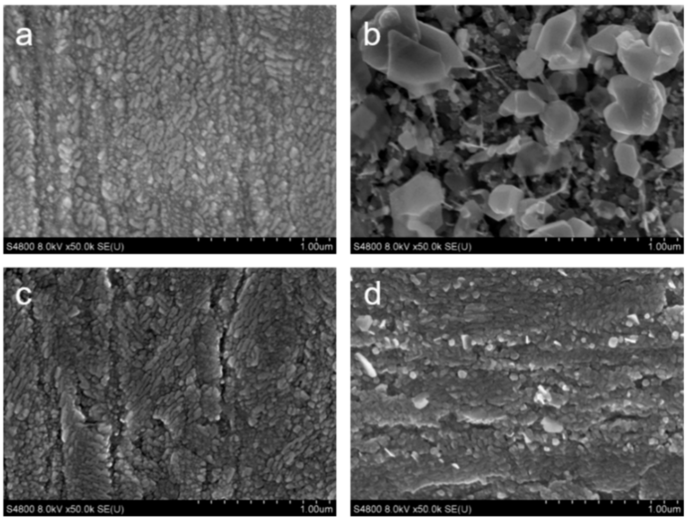

Figure S1. Effect of ultrasound process on the morphology of nanoAg@Cu. SEM images of treated Cu(0) surface (a) and nanoAg@Cu (b) without ultrasound. SEM images of treated Cu(0) surface (c) and nanoAg@Cu (d) after ultrasound for 2 minutes and stand still for 8 minutes in deionized water.

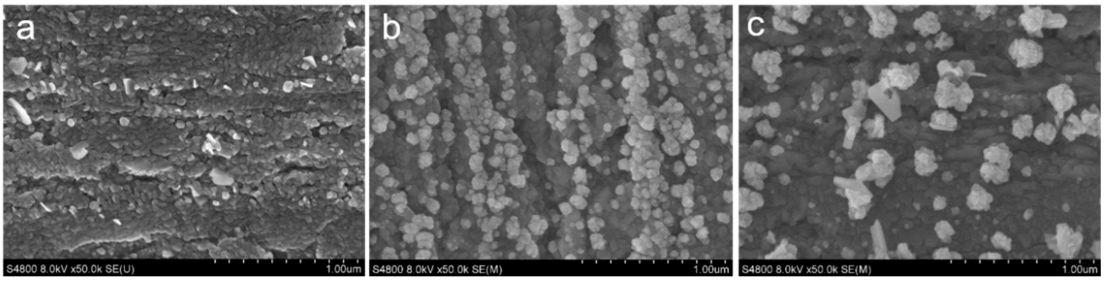

Figure S2. Effect of the adding rate of $AgNO_3$ solution on the formation of nanoAg@Cu. From a to c the adding rates are one-time join (a),120 μL/min (b) and 60 μL/min (c), respectively.

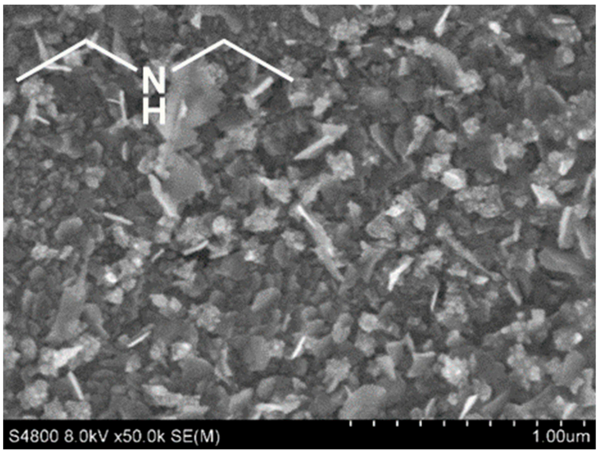

Figure S3. SEM image of nanoAg@Cu obtained by adding diethylamine (DEA) in growth solution.

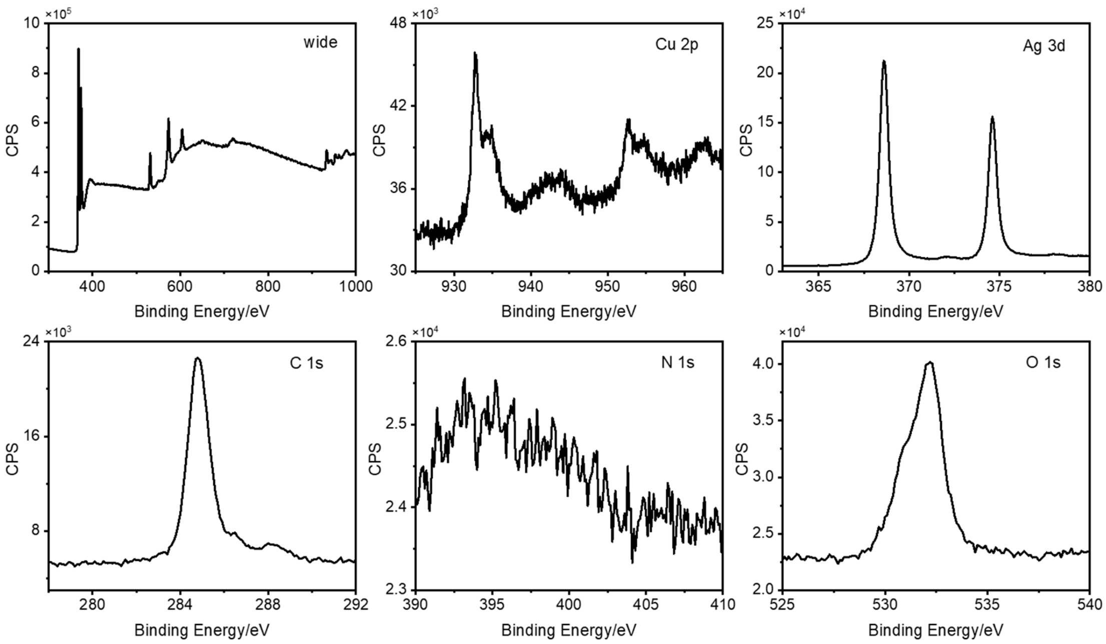

Figure S4. The X-ray photoelectron spectroscopy of nanoAg@Cu tuned by HS-PEG-NH_2.

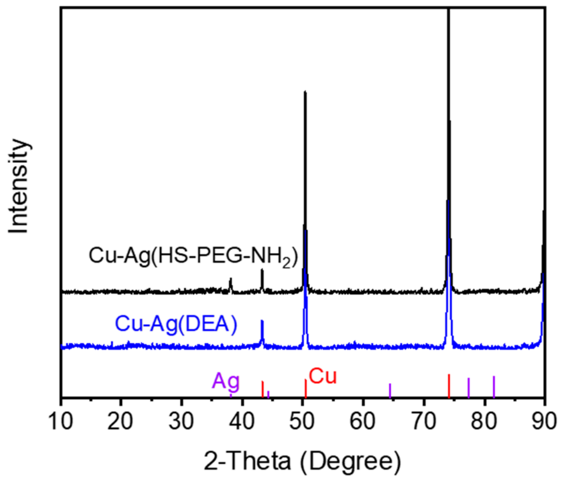

Figure S5. The XRD characteristic of nanoAg@Cu adjusted by DEA and HS-PEG-NH_2.

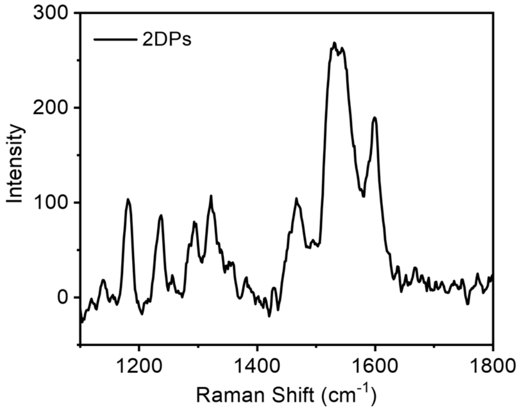

Figure S6. The Raman spectrum of 2DPs.

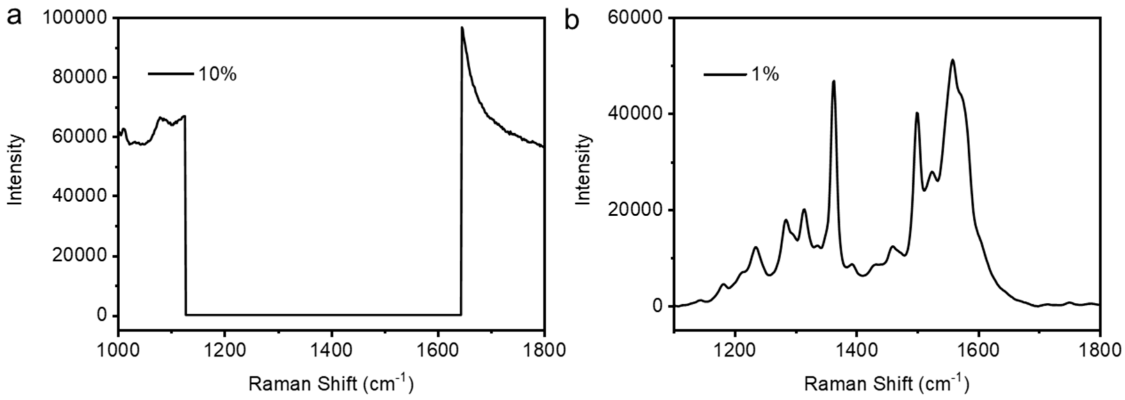

Figure S7. The Raman spectrum of 2DPs on the nanoAg@Cu obtained with the $AgNO_3$ concentration of 5 mM in the presence of 1 mM HS-PEG-NH_2 at laser power of 10 % (a) and 1 % (b).

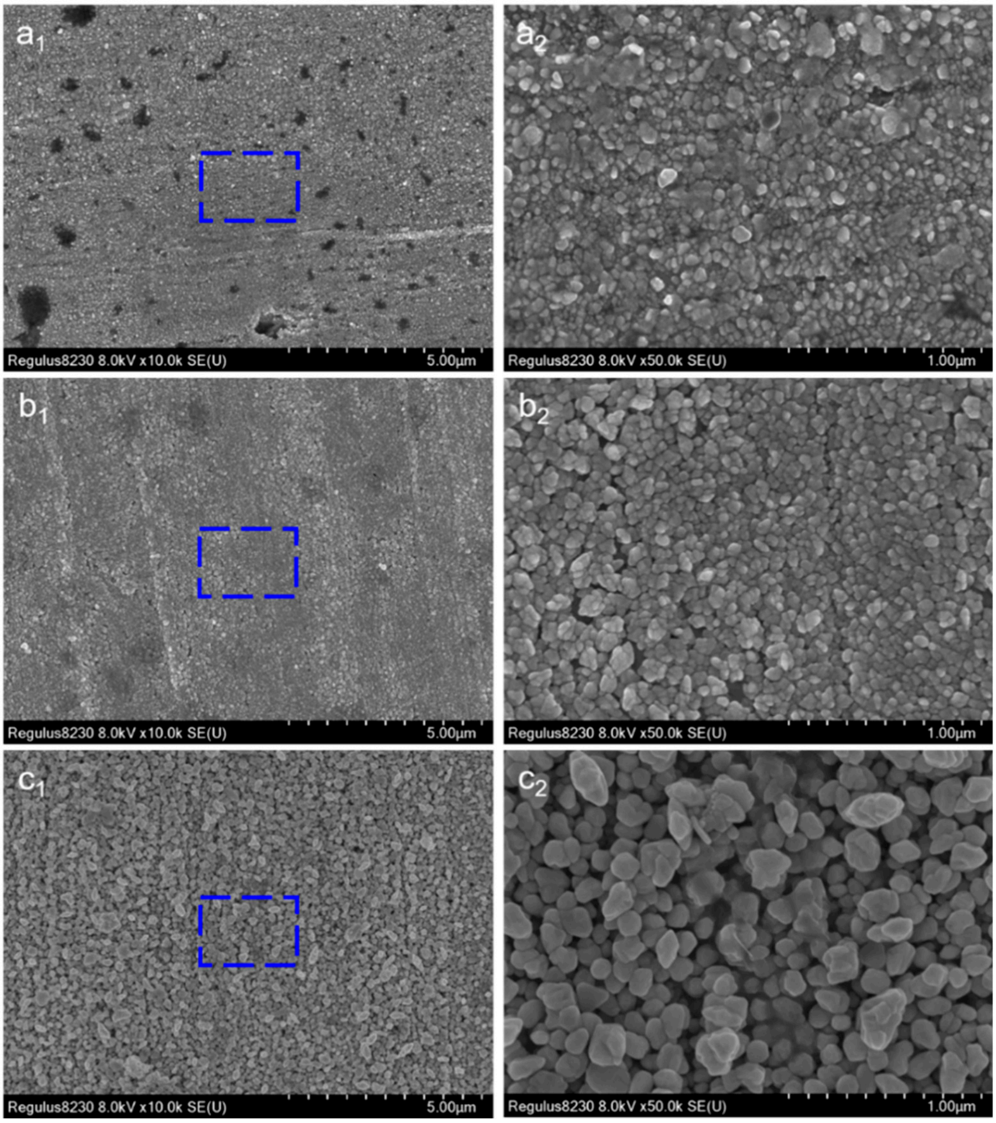

Figure S8. The SEM images of nanoAg@Cu by adjusting with HS-PEG-NH_2 and increasing the concentration of $AgNO_3$. The concentration of $AgNO_3$ are 1 mM (a), 5 mM (b) and 10 mM (c), respectively.

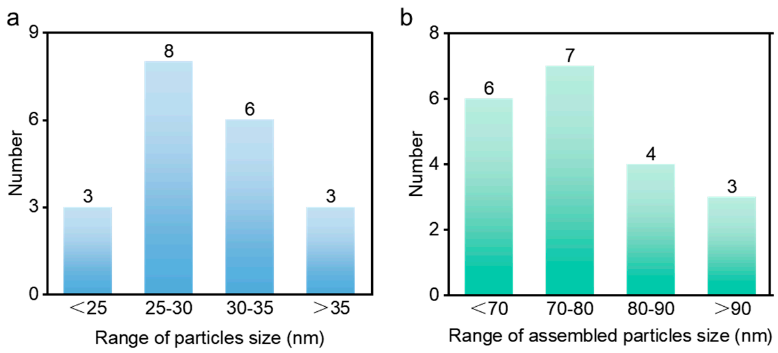

Figure S9. Distribution of diameter of Ag nanoparticles (a) and assembled particles (b) obtained from effecting of 5 mM $AgNO_3$ under the influence of 1 mM HS-PEG-NH_2.

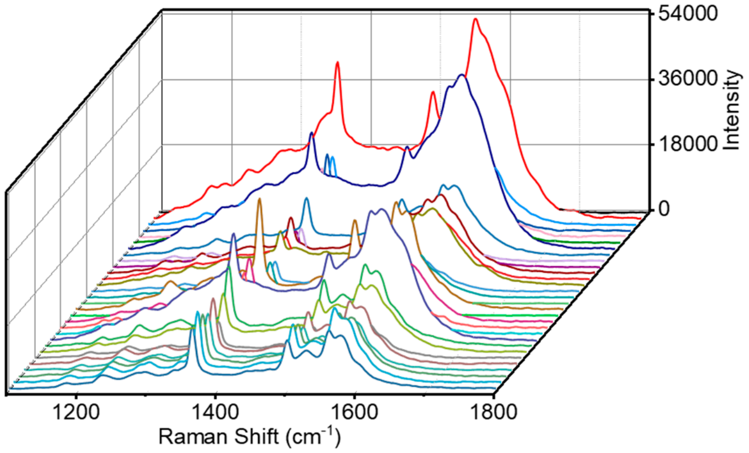

Figure S10. The repeatability test results of the SERS signals from 30 points of one sample with the nanoAg@Cu obtained from 10 mM $AgNO_3$ solution.
